# Magnetic resonance–guided focused ultrasound thalamotomy for tremor

**DOI:** 10.3171/2024.7.FOCVID249

**Published:** 2024-10-01

**Authors:** Jason A. Chen, P. Jason White, G. Rees Cosgrove, John D. Rolston

**Affiliations:** 1 Department of Neurosurgery, Brigham and Women’s Hospital, Boston;; 2 Department of Radiology, Focused Ultrasound Lab, Brigham and Women’s Hospital, Boston; and; 3 Department of Chemistry and Physics, Simmons University, Boston, Massachusetts

**Keywords:** focused ultrasound, Parkinson’s disease, essential tremor, ventralis intermedius nucleus, thalamotomy

## Abstract

Magnetic resonance–guided focused ultrasound (MRgFUS) thalamotomy has emerged as an effective treatment for tremor, particularly in those patients who are excluded from deep brain stimulation. The authors illustrate an example of MRgFUS thalamotomy, targeting the ventralis intermedius nucleus, in a 78-year-old patient with tremor who had features of essential tremor and tremor-predominant Parkinson’s disease. Significant tremor improvement was seen during the procedure. The authors review step-by-step the preoperative considerations, Vim targeting, treatment, and outcomes for this evolving treatment modality.

The video can be found here: https://stream.cadmore.media/r10.3171/2024.7.FOCVID249

## Transcript

### 0:24 Patient History.

This is a 78-year-old man with a 9-year history of debilitating bilateral hand tremor, with features of both essential tremor and Parkinson’s disease. He was refractory to propranolol, primidone, and levodopa. We decided to treat with MRI-guided focused ultrasound (MRgFUS) thalamotomy.^1–3^ Skull density ratio (SDR) was found to be 0.31; although suboptimal, this factor alone does not obviate successful treatment.^4^

### 0:51 Preparation.

As hair can interfere with the ultrasound focusing, we shaved the patient’s head and affixed a stereotactic frame following injection of local anesthetic. The frame is placed low on the head to maximize the number of ultrasound sources available during the procedure. The patient is brought to the MRI scanner, where a membrane helmet is placed. This forms a tight seal with the head. It is filled with cool, degassed water, which completely fills the membrane; final air bubbles are removed by vigorous tapping.

### 1:30 Targeting.

Baseline images are obtained, including a white matter–nulled scan for targeting and a 3D FIESTA scan to define the anterior commissure–posterior commissure (AC-PC) landmarks. Indirect coordinates for the ventral intermediate nucleus (Vim) target are approximately 2 mm above the AC-PC plane, one-quarter of the AC-PC distance anterior to PC, and 14 mm lateral to the midline.^5^ The ultrasound transducer is centered around this target. Automated segmentation and tractography that estimates the dentatorubrothalamic tract (DRTT) assist with target refinement.^6^ A white matter–nulled sequence also helps with direct targeting. The Vim appears slightly hypointense here. We have also observed that the anterior-posterior (AP) position of the target often lines up with an interruption in the internal capsule (red arrow), which we use as a landmark. Internal fiducials are placed afterward. We provide a safety button to the patient that can terminate the sonication.

### 2:46 Alignment.

An alignment sonication at subthreshold power demonstrates targeting accuracy, as here MRI thermometry shows heating at the target. We repeat this in three orthogonal planes. After each sonication, we evaluate for any side effects and perform a neurological exam in which we assess speech, sensation in the fingers and face, and tremor. Here, the patient’s postural tremor has already improved. His intention tremor, though, is still severe. We perform pen-to-paper testing; he cannot even attempt to draw a spiral, and he is severely impaired in trying to trace a straight line.

### 3:54 Treatment Sonications.

We then perform a low-energy treatment sonication at the target, which uses higher energy than the alignments. MRI thermometry shows a higher and more sustained temperature rise. After treatment, the intention tremor has improved; on pen-to-paper, he can now follow the spiral and can more precisely trace a straight line.

We continue to enlarge the lesion with higher energy delivery and additional target points; here, we are slightly anterior and superior. MRI thermometry showed sustained tissue temperature above 54°C–55°C. The sonications proceeded smoothly despite the patient’s low SDR. Testing of the tremor revealed further improvement with subsequent lesioning. The patient reported only transient headache and scalp warmth. Pen-to-paper test shows continued improvement.

The patient is disconnected from the ultrasound apparatus and carefully withdrawn from the MRI. He was discharged to home the same day.

### 5:28 Follow-Up.

We follow up the next day. The patient’s tremor had dramatically improved; he did require a cane given some unsteadiness, which in our experience is typically transient, but otherwise experienced minimal side effects. We obtained an MRI, which demonstrated a 177-mm^3^ lesion with surrounding edema at the targeted Vim. We have seen durable response to at least 5 years of follow-up.^7^ The patient wished to pursue treatment of the right thalamus, which could be done in a staged fashion in 9 months.^8^

## Acknowledgments

We thank our patients and their families, and the focused ultrasound team at Brigham and Women’s Hospital.

## Disclosures

Dr. Chen reported equity in Verge Genomics and Gravity Medical Technology. Dr. Cosgrove reported a clinical trial research grant from Insightec Inc.; and consultant educational fees from Insightec Inc. outside the submitted work.

## Author Contributions

Primary surgeon: Rolston. Assistant surgeon: Chen. Editing and drafting the video and abstract: Rolston, Chen, Cosgrove. Critically revising the work: Rolston, Chen, Cosgrove. Reviewed submitted version of the work: all authors. Approved the final version of the work on behalf of all authors: Rolston. Supervision: Rolston.

## Supplemental Information

### Patient Informed Consent

The necessary patient informed consent was obtained in this study.
